# Validation of the Holmlund-Grooten sub-maximal arm crank ergometer-test for estimating peak oxygen uptake in wheelchair users with Spinal Cord Injury

**DOI:** 10.1371/journal.pone.0344188

**Published:** 2026-04-01

**Authors:** Tobias Holmlund, Wilhelmus Johannes Andreas Grooten

**Affiliations:** 1 Karolinska Institutet, Department of Neurobiology, Care Sciences and Society, Division of Physiotherapy, Huddinge, Sweden; 2 Dalarna University, School of Health and Welfare, Falun, Sweden; Università degli Studi di Milano: Universita degli Studi di Milano, ITALY

## Abstract

**Study design:**

Cross-sectional validity study.

**Objectives:**

To determine the criterion validity, Standard Error of the Measurement (SEM) and Minimal Detectable Change (MDC), of a newly developed submaximal test (Holmlund-Grooten test) for estimating the peak oxygen consumption in wheelchair users with motor-complete Spinal Cord Injury (mcSCI).

**Settings:**

Outpatient rehabilitation centre in Stockholm, Sweden.

**Methods:**

Peak and submaximal oxygen uptake (VO_2_) was measured using indirect calorimetry. A backward linear regression model, including heart rate, power output, and several demographic variables, was used to predict absolute VO_2peak_.

**Results:**

In total, 63 individuals (16 females) with mcSCI were included. The final prediction model included four significant (p < 0.05) variables (sex, injury level, heart rate, power output) that were able to predict absolute VO_2peak_ (adjusted R^2^ = 0.77). The Holmlund-Grooten test showed excellent criterion validity (ICC = 0.88; 95%CI 0.83–0.94), SEM = 0.05 L·min^-1^ and MDC = 0.13 L·min^-1^. No adverse events were reported. We provide an Excel file that calculates VO_2peak_ based on the Holmlund-Grooten model for males and females and tetra- and paraplegia for clinical use.

**Conclusion:**

The Holmlund-Grooten test is a valid submaximal test for estimating VO₂_peak_ in wheelchair users with SCI and can be easily implemented in clinical settings.

## 1. Introduction

Spinal Cord Injury (SCI) is a severe and life-changing medical condition. Individuals with SCI often experience various physical, psychological, and social challenges due to physical impairments, chronic pain, depression, anxiety, and social isolation [1–4]. A motor-complete SCI (mcSCI) contains paralysis from the upper body and down depending on the neurological level of injury; this entails a life in a wheelchair and several consequences that affect many body functions [5,6]. Consequently, it affects the prerequisites for physical activity and reduces physical capacity and peak (VO_2peak_) or maximum oxygen uptake (VO_2max_), and cardiovascular work capacity reduces quickly in the early stages of rehabilitation [7,8]. Subsequently, persons with mcSCI have a higher risk for metabolic syndrome and premature death due to respiratory and cardiovascular diseases (CVD) [9–12]. Low physical activity levels and early weight escalation impact the person’s independence. Previous research has shown that physical activity/exercise and sedentary behaviour are strongly and independently associated with self-perceived health and risk factors for cardiovascular disease [13]. Various strategies are employed to reduce the risk of CVD, including managing different risks and using medications [14]. Conversely, lifestyle changes like physical activity and dietary change are the most common and effective management options alongside pharmacological interventions [14,15]. Consequently, activity, diet and physical capacity need to be better monitored in individuals with SCI, especially mcSCI.

Regularly testing the physical capacity during the rehabilitation of mcSCI has the potential for clinicians and researchers to assess and evaluate the progress and individually adjust the exercise programs [16]. This enables support for individualised exercise programs to improve physical capacity, which has the potential to improve activities of daily living. Moreover, many individuals with mcSCI participate in sports, e.g., Paralympics, and monitoring physical capacity by testing VO_2peak_ over time is a prerequisite for success [8]. Monitoring physical capacity can be performed with maximal or submaximal tests. Since maximal tests require great effort from the individual and expensive laboratory equipment, submaximal tests are mostly used to assess and estimate physical capacity. Commonly used submaximal tests in the able-bodied population are conducted with lower extremity exercise, e.g., Åstrand test [17] or Ekblom Bak test [18], but these tests are not applicable in the SCI population. In individuals with mcSCI, a recent review of the literature showed that maximal tests are more commonly used (n = 105) than submaximal tests (n = 28) [19]. Eerden et al. (2018) showed that these maximal tests have several drawbacks, such as motivational issues and adverse events, e.g., a fall in systolic blood pressure [19]. They also found a large variety in patient characteristics, test objectives, protocols, exercise modes (wheelchair, crank ergometry), and outcome parameters [19]. Furthermore, the review showed various opportunities for applying exercise testing in SCI rehabilitation. Still, the findings did not enable us to describe a preferable test protocol for submaximal testing. For example, there are differences in physiological responses between those with an injury level above or below thoracic level six (T6). The sympathetic system directs blood to the skeletal muscles (T1-5) of the upper and lower body (T6-L2), increasing heart rate (HR) and stroke volume, and increasing respiratory rate, oxygen uptake (T1-T5) and blood pressure [20]. The lack of facilitation from the sympathetic system correspondingly affects the cardiovascular system during exercise with reduced cardiac output, stroke volume and oxygen delivery. The result from this is a lower peak HR, lower peak oxygen consumption (VO₂_peak_) and lower peak power for individuals with a mcSCI above T6 [21]. The altered sympathetic function differs also within individuals with motor-complete injuries. For example, individuals with an injury T2-T5 having higher maximum HR, VO_2peak_ and peak power than those among individuals injured between C5 and T1 [20,21]. Furthermore, those with a motor-compete injury below T6 have an almost normal cardiovascular response for HR, VO₂_peak_, blood pressure and peak power [20,21].

Hence, a clinical test of physical capacity should be submaximal, easily administered without directly assessing oxygen consumption, include arm crank ergometry, and apply to both sexes and different levels of injury. A newly designed test, the Holmlund-Grooten test, tried to endorse all these aspects. The present study aimed to determine the criterion validity and psychometric properties of a newly developed Holmlund-Grooten test for estimating peak oxygen consumption in individuals with mcSCI.

## 2. Materials and methods

Data were collected between 01042012 and 30112015 in which peak VO_2_ data was collected to describe energy expenditure for individuals with mcSCI injury levels C5 – T1 and T7-T12 [22,23].

### 2.1 Recruitment

This cross-sectional study was conducted between 2012 and 2015 at an outpatient rehabilitation clinic in the Stockholm area (Sweden). Participants (n = 63) were recruited through various channels, including advertisements on websites and organisations specific to SCI and through word-of-mouth and referrals from the regional SCI unit. Medication and injury level information was obtained, and data collection procedures were explained. If there were any uncertainties about inclusion or exclusion criteria, the researcher requested permission to consult the attending physician or sought the participant’s help. Inclusion criteria for the study required motor-complete tetraplegia with a level of injury between C5-C8 and American Spinal Injury Association impairment scale (AIS) [24], A + B, or motor-complete paraplegia with a level of injury between T7-T12 and AIS A + B [25]. Participants had to be 18 years old and at least one-year post-injury, with absent or minimal self-reported spasticity on the PENN spasm frequency scale. Exclusion criteria included a known history of coronary artery disease, angina, chronic congestive heart failure, resting hypertension, chronic obstructive pulmonary disease, shoulder pain, or other known conditions that could limit exercise ability. Each participant provided written informed consent, and ethical approval was given by the Stockholm region ethics committee, reference number 2011/1989-31/1.

### 2.2 Oxygen uptake measurements

All testing was performed the same day with rests between the submaximal tests and peak test. In total, 12−14 tests were performed as described in previous papers [22,23]. The participants were asked to refrain from smoking and vigorous activity for 12 hours before testing. A Jaeger Oxycon mobile system (Jaeger Oxycon mobile system, Hoechberg, Germany) was used to collect data for oxygen consumption (VO_2_ and VCO_2_) during the arm cranking test. Participants wore a face mask with a ventilation turbine and a sampling tube to collect breath-by-breath data, which was sent to a portable housing unit to analyse oxygen and carbon dioxide content, which was verified with reference gases and room air before the start of each test. A 3% mean VO_2_ variation between reference gases and room air for each steady-state measurement was accepted [26]. HR data were collected using Polar chest straps. Each test lasted at least six minutes, and the last three minutes of oxygen consumption data were used to calculate absolute litres per minute (L·min^-1^) and relative oxygen consumption and millilitres per bodyweight per minute (mL·kg^-1^·min^-1^) HR. The mobile system was validated for lower oxygen consumption levels before testing to compare the accuracy of the mobile system with a traditional Douglas Bag method. Ten non-injured individuals sat quietly and breathed in the facemask connected to both devices for 10 minutes each. The results showed no significant difference between the two methods, with a mean error of −0.27 range of −0.22 to −0.32 L·min^-1^ and a mean variation of 3% [26]. The use of a portable oxygen consumption system originated from data collected for 12 indoor and outdoor activities where the data for this article were collected [22,27].

#### 2.2.1 Submaximal testing.

The submaximal cardiorespiratory capacity was tested using an arm ergometer (Ergommedic 89E Monark, Sweden) placed on a height-adjustable table to achieve the best shoulder position, i.e., the most comfortable position for each individual. Participants were allowed to use upper-body straps or gloves if they had poor balance or hand function. In congruence with previous submaximal testing protocols [17,18], the submaximal test began with 6 minutes at a low workload of 18–24 Watt for paraplegia and 5–10 Watt for tetraplegia, and Borg RPE 9–12 for all. The workload was then increased to 36–42 Watt for paraplegia and 10–25 Watt for tetraplegia, with Borg RPE 13–14, for at least 6 minutes. A steady state was based on stable HR and RPE and was achieved within 4 minutes at the higher work rates. Since continuous work was performed, steady state was reached even faster at lower power output. The cadence was set at 60 RPM (revolutions per minute). The starting level for power output was based on a physical activity questionnaire (F-FAR) and those who reported aerobic exercise at least two times a week time started on a higher workload [28]. To verify if the workload was correct, the participants were asked to rate their perceived excerption on the Borg RPE scale during the first two minutes. The workload was increased or decreased if the ratings were below or over 9–12 for “low workload” and below or over 13–14 for “high workload” [28].

#### 2.2.2 Peak oxygen test.

The VO_2peak_ test protocol was designed based on international laboratory protocols [29] in previous research [30] and was individualised for each participant. The participants positioned their wheelchairs by the arm ergometer (Ergommedic 89E Monark, Sweden) on a height-adjustable table to achieve optimal shoulder position. Participants with poor hand function were allowed to use an upper-body strap or gloves. The test protocol was individualised based on the participant’s workload and results during the submaximal arm-cranking test. The test began with a 3-minute warm-up with a starting workload based on submaximal testing (Table 1). To avoid early lactate production, the cadence was individually chosen between 70 and 110 RPM for persons with motor-complete paraplegia and 50 and 90 RPM for those with tetraplegia. The resistance was increased every minute for at least three and up to six minutes. During the last 1−3 minutes of testing, individualisation included increasing the cadence instead of resistance and was based on Borg RPE and visual assessment. Verbal encouragement was provided during the test. Oxygen consumption (VO_2_ L·min^-1^) and HR were continuously measured and analysed in ten-second averages. VO_2peak_ was determined based on the highest mean oxygen consumption value for 30 seconds. For the test to be considered eligible as VO_2peak_, participants had to achieve a test time of at least 6 minutes, levelling off despite increased resistance or cadence (RPM), and a Borg RPE score >16 supported by respiratory exchange ratio (RER) or respiratory quotient (RQ) above 1.1 [23].

**Table 1:** The starting workload for the VO_2peak_ test protocol and the individual increase in workload during testing (every minute, for three to six minutes).

Submaximal workload (Watt)	Starting workload (Watt)	Increased workload (Watt)
10-15	10	0.25
20-25	10	0.50
36	24	0.50
42	36	0.75

### 2.3 Submaximal model development

**Fig 1:**
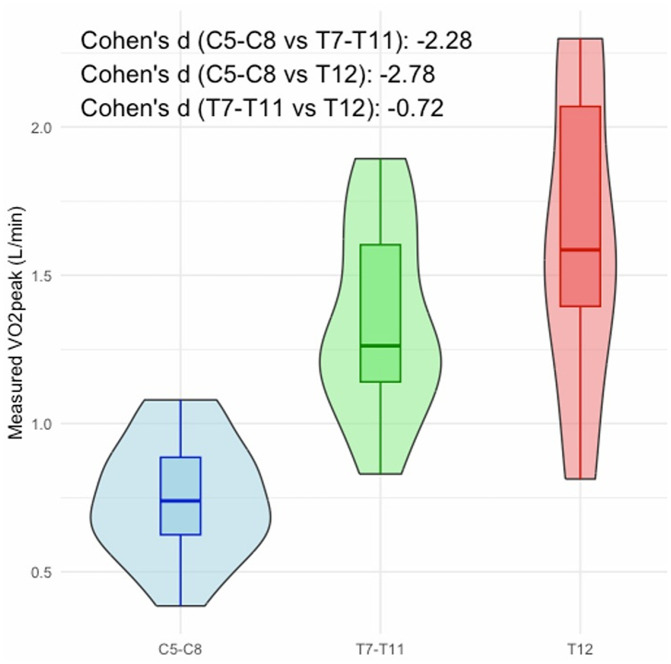
Violin plots for VO2peak (l/min) by injury level (n = 62).

The physiological responses for exercise for individuals with a mcSCI are related to the level of injury; most cervical and thoracic injuries down to T6 have altered physiological responses from the sympathetic nervous system, which need to be considered in the model according to West et al [31]. Correlation analyses were performed, which included sex, age, years since injury, level of injury (continues,) weight, height, and body mass index (BMI). All variables were introduced in a multiple linear regression analysis with VO_2peak_ as the dependent variable. Independent variables were added with backward selection in order of significance. Moreover, injury level was introduced as a continuous variable in automatic linear modelling. However, in a later stage, this variable was split into three categories for best fit by introducing 0 = C5-C8, 1 = T7-T11 and 2 = T12 in the regression model, since the distribution of VO_2peak_ for the different injury levels differed from each other (Fig 1). The distinction between injury levels in paraplegia was further supported by a significant statistically difference (p < .001) in relative VO_2peak_ —18 mL·kg ⁻ ¹·min ⁻ ¹ for T7-T11 and 23 mL·kg ⁻ ¹·min ⁻ ¹ for T12— while body mass and BMI showed no statistically significant differences. Additionally, the VO_2peak_ (L·min^-1^) demonstrated a slight overlap in 95% credible intervals between the T7–11 and T12 groups (mean 1.35; 95% CI 1.23–1.47 and mean 1.62; 95%CI 1.42–1.82, respectively), but not with the C5–C8 group (mean 0.74; 95% CI 0.613–0.864).

There are a few studies including injury levels between T1-T6 [31], indicating that there are few individuals in this category and this study could neither recruit such subjects. Still, we suggest that there is a need for a method for including individuals with these injuries. Individuals with high-level paraplegia (T1-T5) share characteristics with both tetraplegia and lower-level paraplegia in terms of cardiorespiratory control [32]. Level T1-T5 injuries have a different impact on the sympathetic nervous system, which affects HR, blood pressure, and respiratory function. Injuries at these levels provide sympathetic innervation to the heart and upper thoracic region, influencing cardiovascular responses and respiratory mechanics [33,34]. Higher levels (T1–T3) have a more direct impact on HR and upper respiratory muscles, while lower levels (T4–T5) contribute more to vascular tone and some aspects of respiratory function [34]. Overall, high-level paraplegia results in intermediate reductions in cardiac size and function, reflecting some impairment but not as severe as in tetraplegia. Those with injury levels T1-T2 could be categorised as having a cervical injury, as the muscles stimulated by nerves from these areas do not affect VO_2peak_ measurements [32]. Injuries at this level are, however, relatively rare and do functionally not differ that much from injuries at C8-level. Simultaneously, individuals with T3-T6 could be grouped with the T7-T11 group, as the muscles involved in breathing and HR regulation are affected [24]. A test protocol for the Holmlund-Grooten test for wheelchair users is available in S1 File.

### 2.4 Statistics

Both demographic and test data were used in the models. Demographic variables included sex (female/male), age (cont.), years since injury (cont.), level of injury (cont.) weight (kg), height (m), and body mass index (BMI; kg/m2)). From the submaximal tests three variables were extracted: power output (Watt), RPE Borg (6–20) and HR (bmp). Test and demographic data were described in means and standard deviations, min/max, and categorical variables in proportions. Pearson correlation analyses were conducted to examine associations between VO₂_peak_ and potential predictors (heart rate, power output, demographic variables). A backward linear regression model was applied to identify significant predictors of VO₂_peak_. Between-group differences in demographic characteristics (age, height, weight, BMI) and physiological test variables (heart rate, Borg RPE) were examined using one-way analysis of variance (ANOVA). When distributional assumptions of normality or homogeneity of variance were not met, non-parametric Kruskal–Wallis tests were applied as distribution-free alternatives, and Games–Howell procedures were used for post hoc pairwise comparisons due to their robustness to unequal variances and sample sizes. VO₂peak was compared across injury-level groups (C5–C8, T7–T11, T12) using one-way ANOVA, following verification of model assumptions based on residual diagnostics and Levene’s test. To assess the robustness of the findings, complementary Kruskal–Wallis analyses were conducted for VO₂peak as well. Multiple comparisons were adjusted using appropriate familywise error control procedures, and standardized effect sizes (Cohen’s d) were calculated to quantify the magnitude of observed group differences. In addition to the frequentist framework, Bayesian ANOVA employing reference priors was conducted to obtain posterior distributions, from which posterior means and 95% credible intervals (95% CI) were derived, thereby enabling a probabilistic interpretation of between-group differences and strengthening inferential validity.

Missing data for HR (11%) and power output (11%) during the submaximal testing, was handled by imputation using automatic analysis. The analysis included five imputations and used linear regression models for scale variables. The models included no interactions, and the maximum percentage of missing values was set to 100%, while the maximum number of parameters in the imputation model was specified as 100. The model was based on descriptive statistics for participant characteristics and the physiological variables (HR, oxygen uptake, RER) collected during the submaximal and maximal arm crank tests.

All variables were included in a backward multiple linear regression model to predict VO_2peak_. The model added independent variables in order of significance into the VO_2peak_ estimation model using a probability of F = 0.05 for entry and 0.10 for removal. The model was tested for the assumption that there is a difference in VO_2peak_ and HR between tetraplegics and paraplegics and that VO_2peak_ is normally distributed. To validate the predictive accuracy of the Holmlund-Grooten submaximal VO_2peak_ test, we employed a cross-validation approach. This method helps assess the model’s generalisability and mitigate the risk of overfitting. The dataset was randomly divided into five subsets, each comprising about 80% of the total sample. These subsets were used to build separate linear regression models including the same variables as in the final prediction model. Each model was trained on the 80% subset (training data) and then validated on the remaining 20% of the data (validation data). To assess the risk of overfitting, we compared the correlation values between the training data and the validation data [35]. Normality of residuals was assessed using the Shapiro-Wilk test, and homoscedasticity was evaluated using the Breusch-Pagan test for heteroscedasticity. The goodness of fit was evaluated using standard metrics such as R-squared, Adjusted R-squared, and the F-statistic. Additional error metrics such as Mean Squared Error (MSE), Root Mean Squared Error (RMSE) and Mean Absolute Error (MAE) were calculated to assess the model’s accuracy. The variance inflation factor (VIF) was used to measure the degree of multi-collinearity in the regression model, where higher VIF values indicate a higher degree of multi-collinearity. Beta-coefficients were interpreted as small (0−.29), medium (.30−.49) and large (>0.50) associations [36]. Scatter plots of the included variables were used to inspect the data visually and to detect outliers and influential data points that could compromise the results.

Intraclass correlation coefficients (ICC) with 95% confidence intervals were used to assess agreement between predicted and measured VO₂_peak_. We selected a two-way mixed-effects model with absolute agreement, appropriate when the same raters (or measurement method) are used across all subjects and no generalization to other raters is intended. This model assumes that the raters are fixed, and the subjects are randomly sampled, which aligns with the design of our study. Absolute agreement was chosen to capture both consistency and systematic differences between predicted and measured values, as recommended by Trevethan (2017) and Koo & Li (2016) [37,38].

As a final step, a visual analysis of the Bland-Altman plot [39] was used to reveal a possible systematic error between the predicted and measured VO_2peak_. In a Bland-Altman plot, the difference between predicted and measured VO_2peak_ is plotted against the mean of the predicted and measured VO_2peak_ and the mean difference and the limits of agreement (LoA); mean ± 1.96SD. All statistical analyses used a significance level of p ≤ 0.05 (two-tailed) and were performed using SPSS 24.0 software and MS Excel.

## 3. Results

### 3.1 Participants

Sixty-three participants performed the VO_2peak_ test; however, one participant was excluded (tetraplegia) due to an extreme value of VO_2peak_ (1.60 L·min^-1^) and a HR over 150 beats per minute (bpm). The demographics of the participants are shown in Table 2. In total, 16 females and 46 males participated in the study (*n* = 62). Of these, 37 (59%) had motor-complete paraplegia, and 25 had motor-complete tetraplegia (41%). The age ranged between 20 and 71 years (Mean 42.45 SD 12.4).

**Table 2:** Demographics and VO_2_ data.

	TETRAPLEGIA C5-8 (n = 25)		PARAPLEGIA T7-T11 (n = 27)		PARAPLEGIA T12 (n = 10)		TOTAL GROUP (n = 62)	
Sex (female%)	28%		26%		30%		27%	
	Mean (SD)	Range (min-max)	Mean (SD)	Range (min-max)	Mean (SD)	Range (min-max)	Mean (SD)	Range (min-max)
**Age (years)**	41.8 (13.8)^a^	20-69	42.7 (11.4)	23-71	37.3 (9.5)	20-69	42.3 (12.3)	20-71
**Body height (m)**	1.78 (0.09)	1.65-1.95	1.76 (0.10)	1.58-1.93	1.80 (0.10)	1.65-1.95	1.78 (0.10)	1.58-1.95
**Weight (kg)**	66.0 (12.6)	43.0-92.0	73.8 (13.8)	50.5-102.0	70.5 (16.9)	46.7-100.0	70.1 (14.9)	43.0-102.0
**BMI**	20.6 (3.5)^a^	14.9-26.9	23.7 (3.2)	17.5-29.6	20.6 (3.0)	14.9-26.9	22.1 (3.4)	14.9-29.6
**Years since injury**	15.2 (11.1)	3-38	17.6 (11.)	2-42	10.1 (7.7)	2-27	15.4 (11.2)	2-42
***Submax (measured)***								
**VO**_**2**_ (L·min^-1^)	.55 (0.09)	.37-0.68	.86 (0.14)	.47-1.18	.87 (0.12)	.66-1.18	.74 (.20)	.37-1.18
**Power output (Watt)**	20 (6)^a,b^	10-42	40.0 (3.8)	24-42	40.0 (2.9)	36-42	32.0 (10.8)	10-42
**Heart rate (bpm)**	94.3 (16)^a,b^	70-130	124 (22)	92-162	127 (22)	92-162	112 (24)	70-162
**BORG RPE**	15 (1.8)	10-17	13 (2)	9-17	13 (3)	9-17	14 (2)	9-18
***VO***_***2peak***_ ***(measured)***								
**VO**_**2**_ **(L·min**^**-1**^)	.74 (0.26)^a,b^	.39-1.60	1.35 (.39)	.83-1.89.	1.62 (.51)	81-2.30	1.16 (0.47)	.39-2.30
**VO₂**_**peak**_ **(mL·kg ⁻ ¹·min ⁻ ¹)**	11.3 (2.5)^a,b^	7.6-16.7	18.3 (2.6)^c^	13.9-25.6	22.8 (4.0)	17.4-30.1	16.2 (5.2)	7.6-30.1
**Heart rate (bpm)**	107 (17)^a,b^	61-128	171(16)	131-198	182 (14)	152-195	145 (38)	61-198

BMI = body mass index, bpm = beats per minute, ^a^ = Significant (p < 005) group difference Tetraplegia vs Paraplegia(T7-T11), ^b^ = Significant (p < 005) group difference Tetraplegia vs Paraplegia (T12),^c^ = Significant (p < 005) group difference Paraplegia (T7-T11) vs Paraplegia (T12).

### 3.2 Regression model for estimation of VO_2peak_

The initial model included ten variables: age, injury level, sex, power output, HR and Borg RPE (high workload), times since injury, body weight, height, and BMI. The final correlation matrix showed that four (two categorical and two continuous) variables: injury level, sex, HR and power output (Watt) were significantly (p < 0.05) related to the outcome of VO_2peak_ (Equation and Table 3).

**Table 3:** Unstandardised and standardised coefficients for the included variables, Standard error, t-statistics, p-values, and 95% CI for B and Beta. Collinearity statistics, Tolerance, and Variance inflation factor (VIF).

	Unstandardised Coefficient		Standardised Coefficient	t	Sig.	95% Confidence Interval for B		Collinearity Statistics	
	B	Std. Error	Beta			Lower Bound	Upper Bound	Tolerance	VIF
**(Constant)**	0.674	0.154		4.368	<0.001	0.365	0.983		
**Sex (0 = female, 1 = male)**	0.299	0.072	0.284	4.238	<0.001	0.155	0.443	0.821	1.217
**Injury level (0, 1, 2)**	0.278	0.066	0.430	4.221	<0.001	0.146	0.410	0.403	1.161
**Heart rate (bpm)**	−0.006	0.001	−0.325	−4.209	<0.001	−0.009	−0.003	0.645	1.551
**Power output (Watt)**	0.024	0.005	0.552	4.791	<0.001	0.014	0.034	0.821	1.217

Injury-level: 0=tetraplegia C5-C8; 1= paraplegia: T7-T11; 2= paraplegia: T12.

Bmp = beats per minute.

**Equation**: Final regression model for estimating VO_2peak_ from submaximal arm crank ergometry.


$${\text{VO}}_{\text{2peak}}=\text{ 0}.\text{674022 }+\;(\text{Sex }*\text{ 0}.\text{299016})+\;(\text{Injury level }*\text{ 0}.\text{278286})-(\text{HR }*\text{ 0}.\text{006232})+\;(\text{Watt }*\text{ 0}.\text{023712})$$

*The model’s categorical variables are sex (female = 0; male = 1) and injury level: tetraplegia C5-C8 = 0; paraplegia T7-T11 = 1; T12 = 2. Heart rate (HR): Tetraplegia 70–130 bpm. Paraplegia 92–162 bpm. Power output: Tetraplegia 10–25 Watt; Paraplegia 36–42 Watt.*

**Fig 2:**
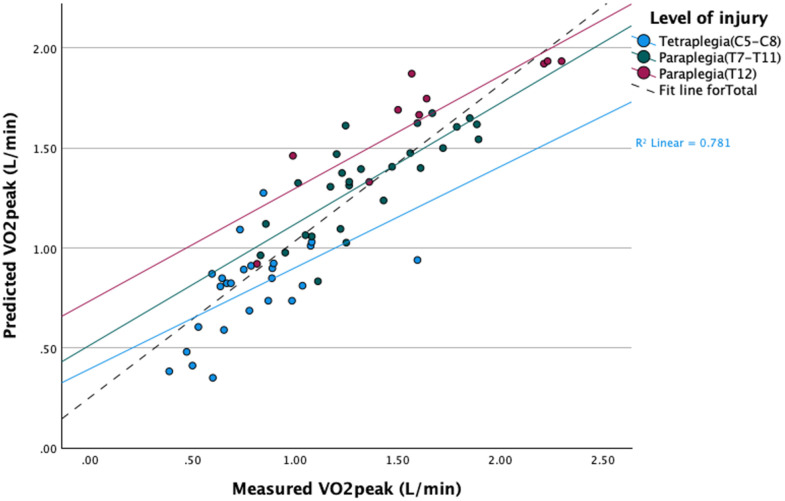
Scatterplot of measured VO_2peak_ plotted against predicted VO_2peak_. Fit line for total group (R^2^ = 0.78), blue for tetraplegia, green for paraplegia (T7-T11), red for paraplegia (T12).

The multiple linear regression model showed a strong fit, indicating that approximately 78.1% of the variance in VO_2peak_ was explained by the predictors (adjusted R^2^ = .766), while the standard error of the estimate (SEE) was calculated to be 0.22 VO_2_ (L·min^-1^). Collinearity diagnostics showed no multicollinearity concerns, with all VIF values below 3.4 and tolerance values above.29, suggesting that the predictors were not highly correlated. All predictors were statistically significant (*p* < .001), indicating that each variable made a meaningful contribution to predicting VO_2peak_. Sex and injury level positively affected VO_2peak_, whereas HR had a negative association. Power output (Watts) had a positive and significant impact on VO₂_peak_. The mean squared error of the model was 0.171 L·min^-1^ and the root mean squared error was 0.216 L·min^-1^. The Shapiro-Wilk test for normality of residuals yielded a *W* = .987 and *p* = .762, indicating that the residuals were normally distributed. The Breusch-Pagan test for heteroscedasticity was non-significant (*p* = .309), confirming that the residuals were homoscedastic. Fig 2 shows scatterplots between estimated and measured VO_2peak_ for the total group and the tetra- and paraplegics separately.

### 3.3 Agreement estimates

**Fig 3:**
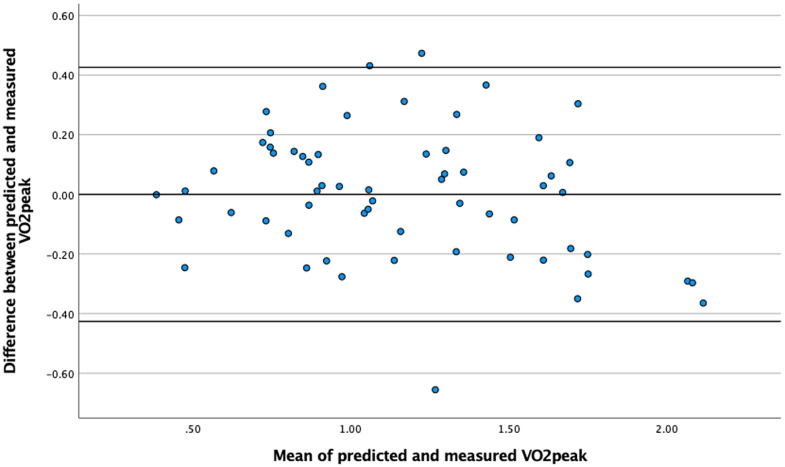
Scatter plot of the difference between predicted and measured against the mean of predicted and measured VO_2peak_. Mean difference −0.00, limits of agreement (LOA) −0.436; + 0.436.

In relative terms, the Intraclass Correlation Coefficient (*ICC*) of.879 implies a good agreement between the measured and the predicted values, with a 95% confidence interval of.834 and.937. In absolute terms, the SEM for VO_2_ was calculated to be 0.046 L·min^-1^ and the MDC to 0.128 L·min^-1^. This suggests that changes in cardiorespiratory fitness can be detected with a subtle change of 0.13 L·min^-1^. As a final step, the Bland-Altman plot showed no signs of systematic errors (Fig 3).

### 3.4 Model validation

To evaluate the performance of our prediction model for VO_2peak_, we employed a cross-validation where we created a regression analysis on approximately 80% randomly chosen participants from the full dataset. The cross-validation models were highly comparable with the full model (R = .884). Consistent high correlations across all datasets and somewhat higher correlation values for the validation data compared to the training data indicate that the models generalise well and are not overfitting (Table 4). Scatterplots of all five sample models are presented in S2 File.

**Table 4:** Cross validation (5 samples) trained on 80% of the data and validated on 20% of the data.

Sample n = 62	R (Training Data)	Standard Error (Training Data)	Correlation with Measured VO_2peak_ (Training Data)	Correlation with Measured VO_2peak_ (Validation Data)
**1**	.877	0.222	.877 (n = 46)	.907 (n = 16)
**2**	.876	0.234	.876 (n = 47)	.889 (n = 15)
**3**	.878	0.225	.878 (n = 55)	.945 (n = 7)
**4**	.859	0.234	.859 (n = 48)	.929 (n = 14)
**5**	.872	0.227	.872 (n = 51)	.946 (n = 11)

R = Explained variance.

VO_2peak_ = Peak oxygen uptake.

### 3.5 Clinical use

An Excel sheet for easy calculating VO_2peak_ in absolute terms L·min^-1^ and relative terms mL·kg^-1^·min^-1^ for wheelchair users with tetraplegia C5-C8 (T1-T2), paraplegics T7-T11 (T3-T6), and T12 for clinical use is available as S1 File. The test leader needs information about three individual variables: sex, injury level and body weight and two test variables: HR and power output (Watt). Additional calculators for the transformation from RPM and kpa to Watt and the calculation of mean HR and mean Watt in the protocol are available in the Excel sheet.

## 4. Discussion

This study aimed to test the validity of a new clinical test – the Holmlund-Grooten test – for estimating VO_2peak_ using submaximal arm crank ergometry for individuals with mcSCI. The test showed good validity as evidenced by significant predictors, a high predictive accuracy and consistent performance across different subsets. The results showed that sex, injury level, HR and power output were significantly related to the outcome of VO_2peak_. The final model explained 77% of the variation in VO_2peak_ and had a standard error estimate of 0.22 VO_2_ L·min^-1^. The validity estimates of the model were also assessed, showing good agreement (ICC = .879) between the measured and predicted values and a minimal detectable change of 0.13 VO_2_ L·min^-1^, suggesting that changes in cardiorespiratory fitness can be detected with a subtle change in VO_2peak_.

### 4.1 Model variables

Previous submaximal tests were not clinically applicable due to the measurement of oxygen uptake [19]. In contrast, the Holmlund-Grooten submaximal test only needs a calibrated arm ergometer bike, heart monitor and no other advanced equipment. This shows that the Holmlund-Grooten test is straightforward to administer and is appropriate for clinical settings equipped with a calibrated arm ergometer bicycle for predicting absolute VO_2peak_. This test is inspired by the Åstrand leg ergometer bike, in which age, sex, HR and power output are included [17]. In addition to the variables in the Åstrand test, the Holmlund-Grooten test included injury level.

The finding that Sex had a significant association with absolute VO_2peak_, with males having higher values than females is consistent with previous research [7]. Moreover, injury level was significantly (positively) correlated to VO_2peak_ and also consistent with previous research [7,8]. Individuals with tetraplegia typically have lower absolute VO_2peak_ values than those with paraplegia due to paralysis affecting muscle mass, the respiratory muscles and the lack of response from the sympathetic nervous system.

This study found that power output (positively) and HR (negatively) correlated with VO_2peak_. Individuals with higher VO_2peak_ have a lower HR at a fixed submaximal workload than those with a lower VO_2peak_. Previous studies on SCI have shown that HR during submaximal testing significantly decreased after just seven days of cardiorespiratory exercise [40]. Fukuoka et al. (2006) showed that 60 days of aerobic exercise decreased HR by 15% from 123 beats/min to 105 beats/min, although the submaximal workload increased from 32 W to 40 W [40]. The authors referred to a significant increase in stroke volume as a possible physiological adaptation since stroke volume increased from 88.1 mL to 106.4 mL after 60 days of aerobic exercise. Koppo et al. (2004) described that the improvement could be explained by increased mitochondrial density, oxidative enzyme activity and concentration, tissue capillary density, and muscle oxygen perfusion, which facilitate oxidative metabolic recovery after sustained exercise [41]. The study by Murray et al., reported a 30–38% lower VO_2peak_ for individuals with SCI compared to those without SCI, showing that untrained individuals with a mcSCI have reduced recovery VCO_2_ off-kinetics efficiency transition constants after aerobic exercise compared to trained individuals with SCI [42]. It’s essential to remember that the upper-body muscles are more susceptible to early fatigue and exhaustion. This could limit cardiorespiratory capacity and lengthen recovery time, particularly for individuals with a cervical level of injury. This further illuminates the importance of early adaptations of VO_2_ kinetics, which will positively affect exercise tolerance and may impact daily living activities. All in all, these studies indicate that the Holmlund-Grooten Test could be suitable for monitoring changes in cardio-respiratory capacity in wheelchair users with SCI.

### 4.2 Validity

The present criterion validity study showed high predictive accuracy of a new submaximal test against a “gold standard” of maximal testing of VO_2peak_. The Holmlund-Grooten test is the first test for predicting absolute VO_2peak_ for persons with mcSCI without direct oxygen consumption measurements during submaximal testing. The prediction model by Totosy de Zepetnek et al. [43], (6MAT) included measuring oxygen uptake during the submaximal testing for predicting relative VO_2peak_ for individuals with an SCI. The 6MAT showed a SEE of 3.86 mL·kg^-1^·min^-1^ for paraplegia and 2.41 mL·kg^-1^·min^-1^ for tetraplegia. Recalculating our data from absolute to body weight-related oxygen uptake, the Holmlund-Grooten test has somewhat lower SEE for paraplegia 3.18 mL·kg^-1^·min^-1^ and slightly higher compared to tetraplegia 2.57 mL·kg^-1^·min^-1^. Moreover, compared to the tests by Åstrand [17] and Ekblom Bak, our model showed a similar variance of 78% compared to Åstrand [44] and somewhat lower compared to 82% by Ekblom Bak [18]. The SEE for absolute oxygen uptake was 0.22 compared to 0.33 L·min^-1^ in the study of Ekblom Bak, and this could be explained by the difference in VO_2peak_ (L·min^-1^), which was more than doubled in the Ekblom Bak cohort [18]. The minimal detectable change for the Holmlund-Grooten test was 0.13 L·min^-1^. Two intervention studies, Holm et al (2024) and Lindberg et al. (2012), showed that individuals with mSCI increased absolute VO_2peak_ by 0.15 and 0.25 L·min^-1^ during rehabilitation, respectively, indicating sufficient accuracy of the Holmlund-Grooten test to detect such changes [45,46].

### 4.3 Cross-validation

The cross-validation of the Holmlund-Grooten submaximal VO_2peak_ test indicated a relatively high predictive accuracy and generalisability [47]. The consistently high correlation values between the predicted and measured VO_2peak_ values across both the training and validation datasets indicate that the models are robust and not overfitting [48]. Specifically, the higher correlation values observed in the validation data compared to the training data suggest that the models perform well on unseen data [35]. The use of cross-validation not only enhanced the robustness of the model but also provided a more reliable estimate of its performance on new data [48,49].

### 4.4 Strengths and limitations

Among the strengths of this study, the submaximal test was found to be easy-administered and safe. Both females and males with mcSCI were included, covering a variety of neurological injury levels that incorporate different sympathetic responses to physical activity. Moreover, the number of participants enabled to provide clinically valuable data for these subgroups. However, individuals with a lesion between T1 and T6 were not included in the data collection since this group is very small. One explanation could be that the spinal cord is protected by the rib cage, a structure with biomechanically robust properties and little mobility. Despite the relatively good performance of the models, there are some limitations to consider. The SEE was found to be proportionally larger compared to VO_2peak_ measures in non-injured populations, possibly due to the large heterogeneity in our mcSCI population. The sample size may have limited the generalisability of the findings to larger and more diverse populations. Additionally, the predictors used in the models, such as power output, heart rate, sex, and injury level, may not have captured all the factors influencing VO_2peak_. As a final comment, the test-retest reliability of the Holmlund-Grooten test has not been established. However, preliminary data from a different sample including seven individuals with SCI revealed an ICC of 0.983 för L·min^-1^, and 0.981 mL·kg^-1^ min^-1^ indicating good reliability.

### 4.5 Clinical implications and future research

The Holmlund-Grooten test predicts VO_2peak_ from submaximal arm crank ergometry and could be used for monitoring changes over time both in and outside a rehabilitation setting. The study’s findings could be used for developing personalised exercise and rehabilitation programs to monitor, evaluate and improve their cardiorespiratory fitness levels in individuals with SCI. This could guide exercise prescription, allowing for more effective and safe exercise training programs. Moreover, monitoring cardiorespiratory fitness levels could be a motivating factor for adherence to exercise programs in a long-term perspective. However, additional research is required. Besides testing the feasibility of the test, future research should examine different types of reliability, such as the test-retest reliability as well as the intra- and interrater reliability. It is also important to explore additional predictors in larger sample sizes to further validate and refine the models. Moreover, it is important to test the construct validity of the test, i.e., the degree to which the scores are consistent regarding relationships to scores of other instruments, or differences between relevant groups (discriminative validity) [50]. Longitudinal studies could also provide insights into the responsiveness, i.e., the stability of the models over time, and their applicability in monitoring changes in cardiovascular fitness. Finally, due to our relatively strict inclusion and exclusion criteria, clinicians should be aware that the Holmlund-Grooten test should not be generalised to persons with AIS D.

### 4.6 Conclusions

This study showed high criterion validity of a newly developed Holmlund-Grooten submaximal test for estimating the peak oxygen uptake in individuals with mcSCI. A calculation sheet for individual estimation of VO_2peak_ in L·min^-1^ is available for clinical usage. There is need for a further validation of the test and to establish the reliability.

## Supporting information

S1 File Calculation sheet. (XLSX)

S2 File Validation checks. (DOCX)
