# Supplementary material for: Validation of the Holmlund-Grooten sub-maximal arm crank ergometer-test for estimating peak oxygen uptake in wheelchair users with Spinal Cord Injury
Source: PLoS One. 2026 Apr 1;21(4):e0344188. doi: 10.1371/journal.pone.0344188 (PMC13042776; doi:10.1371/journal.pone.0344188)

Cross-Validation Procedure

To validate the predictive accuracy of the Holmlund-Grooten submaximal VO2 peak test, we employed a cross-validation approach. This method helps assess the model's generalisability and mitigate the risk of overfitting. The dataset was randomly divided into five subsets, each comprising 80% of the total sample. These subsets were used to build separate linear regression models using the following predictors: Watt, Heart Rate, Gender, and Injury Level. The dependent variable was the measured VO2 peak (L/min). Each model was trained on the 80% subset (training data) and then validated on the remaining 20% of the data (validation data). To assess the risk of overfitting, we compared the correlation values between the training data and the validation data.

**Sample one**

| **Model 1 Summary** | | | | | | | | | |
| --- | --- | --- | --- | --- | --- | --- | --- | --- | --- |
| Model | R | R Square | Adjusted R Square | Std. Error of the Estimate | Change Statistics | | | | |
|  |  |  |  |  | R Square Change | F Change | df1 | df2 | Sig. F Change |
| 1 | .877^a^ | .769 | .746 | .22175 | .769 | 34.106 | 4 | 41 | <.001 |
| a. Predictors: (Constant), Injury level, gender, HR, Watt | | | | | | | | | |

| **Coefficients^a^** | | | | | | |
| --- | --- | --- | --- | --- | --- | --- |
| Model 1 | | Unstandardized Coefficients | | Standardized Coefficients | t | Sig. |
|  |  | B | Std. Error | Beta |  |  |
| 1 | (Constant) | .546 | .173 |  | 3.161 | .003 |
|  | Watt | .024 | .005 | .566 | 4.357 | <.001 |
|  | Heart rate | -.005 | .002 | -.273 | -3.008 | .004 |
|  | Gender | .260 | .085 | .254 | 3.048 | .004 |
|  | Injury level | .256 | .072 | .419 | 3.586 | <.001 |
| a. Dependent Variable: Measured VO_2peak_ (L/min) | | | | | | |

| **Correlations** | | | | |
| --- | --- | --- | --- | --- |
| Sample1 | | | Measured VO_2peak_ (L/min) | Predicted sample 1 |
| .00 | Measured VO_2peak_ (L/min) | Pearson Correlation | 1 | .907^**^ |
|  |  | Sig. (2-tailed) |  | <.001 |
|  |  | N | 16 | 16 |
|  | Predicted sample 1 | Pearson Correlation | .907^**^ | 1 |
|  |  | Sig. (2-tailed) | <.001 |  |
|  |  | N | 16 | 16 |
| 1.00 | Measured VO_2peak_ (L/min) | Pearson Correlation | 1 | .877^**^ |
|  |  | Sig. (2-tailed) |  | <.001 |
|  |  | N | 46 | 46 |
|  | Predicted sample 1 | Pearson Correlation | .877^**^ | 1 |
|  |  | Sig. (2-tailed) | <.001 |  |
|  |  | N | 46 | 46 |
| **. Correlation is significant at the 0.01 level (2-tailed). | | | | |

**Sample 2**

| **Model 2 Summary** | | | | | | | | | | |
| --- | --- | --- | --- | --- | --- | --- | --- | --- | --- | --- |
| Model | R | R Square | Adjusted R Square | Std. Error of the Estimate | Change Statistics | | | | | |
|  |  |  |  |  | R Square Change | F Change | df1 | df2 | Sig. F Change |  |
| 1 | .876^a^ | .767 | .745 | .23377 | .767 | 34.532 | 4 | 42 | <.001 |  |
| a. Predictors: (Constant), Injury level, gender, HR, Watt | | | | | | | | | | |

| **Coefficients^a^** | | | | | | |
| --- | --- | --- | --- | --- | --- | --- |
| Model 2 | | Unstandardized Coefficients | | Standardized Coefficients | t | Sig. |
|  |  | B | Std. Error | Beta |  |  |
| 1 | (Constant) | .706 | .184 |  | 3.829 | <.001 |
|  | Watt | .017 | .007 | .357 | 2.348 | .024 |
|  | Heart rate | -.005 | .002 | -.265 | -2.801 | .008 |
|  | Gender | .370 | .085 | .361 | 4.330 | <.001 |
|  | Injury level | .322 | .081 | .509 | 3.963 | <.001 |
| a. Dependent Variable: Measured VO_2peak_ (L/min) | | | | | | |

| **Correlations** | | | | |
| --- | --- | --- | --- | --- |
| Approximately 80% of the cases (SAMPLE) | | | Measured VO_2peak_ (L/min) | Predicted sample 2 |
| Not Selected | Measured VO_2peak_ (L/min) | Pearson Correlation | 1 | .889^**^ |
|  |  | Sig. (2-tailed) |  | <.001 |
|  |  | N | 15 | 15 |
|  | Predicted sample 2 | Pearson Correlation | .889^**^ | 1 |
|  |  | Sig. (2-tailed) | <.001 |  |
|  |  | N | 15 | 15 |
| Selected | Measured VO_2peak_ (L/min) | Pearson Correlation | 1 | .876^**^ |
|  |  | Sig. (2-tailed) |  | <.001 |
|  |  | N | 47 | 47 |
|  | Predicted sample 2 | Pearson Correlation | .876^**^ | 1 |
|  |  | Sig. (2-tailed) | <.001 |  |
|  |  | N | 47 | 47 |
| **. Correlation is significant at the 0.01 level (2-tailed). | | | | |

**Sample 3**

| **Model 3 Summary** | | | | | | | | | |
| --- | --- | --- | --- | --- | --- | --- | --- | --- | --- |
| Model | R | R Square | Adjusted R Square | Std. Error of the Estimate | Change Statistics | | | | |
|  |  |  |  |  | R Square Change | F Change | df1 | df2 | Sig. F Change |
| 1 | .878^a^ | .771 | .753 | .22509 | .771 | 42.066 | 4 | 50 | <.001 |
| a. Predictors: (Constant), Injury level, gender, HR, Watt | | | | | | | | | |

| **Coefficients^a^** | | | | | | |
| --- | --- | --- | --- | --- | --- | --- |
| Model 3 | | Unstandardized Coefficients | | Standardized Coefficients | t | Sig. |
|  |  | B | Std. Error | Beta |  |  |
| 1 | (Constant) | .579 | .162 |  | 3.569 | <.001 |
|  | Watt_ | .023 | .005 | .565 | 4.526 | <.001 |
|  | Heart rate | -.005 | .002 | -.278 | -3.250 | .002 |
|  | Gender | .311 | .076 | .302 | 4.069 | <.001 |
|  | Injury level | .243 | .070 | .379 | 3.448 | .001 |
| a. Dependent Variable: Measured VO_2peak_ (L/min) | | | | | | |

| **Correlations** | | | | |
| --- | --- | --- | --- | --- |
| Sample 3 | | | Measured VO_2peak_ (L/min) | predicted_dec3 |
| .00 | Measured VO_2peak_ (L/min) | Pearson Correlation | 1 | .945^**^ |
|  |  | Sig. (2-tailed) |  | .001 |
|  |  | N | 7 | 7 |
|  | Predicted sample 3 | Pearson Correlation | .945^**^ | 1 |
|  |  | Sig. (2-tailed) | .001 |  |
|  |  | N | 7 | 7 |
| 1.00 | Measured VO_2peak_ (L/min) | Pearson Correlation | 1 | .878^**^ |
|  |  | Sig. (2-tailed) |  | <.001 |
|  |  | N | 55 | 55 |
|  | Predicted sample 3 | Pearson Correlation | .878^**^ | 1 |
|  |  | Sig. (2-tailed) | <.001 |  |
|  |  | N | 55 | 55 |
| **. Correlation is significant at the 0.01 level (2-tailed). | | | | |

**Sample 4**

| **Model 4 Summary** | | | | | | | | | |
| --- | --- | --- | --- | --- | --- | --- | --- | --- | --- |
| Model | R | R Square | Adjusted R Square | Std. Error of the Estimate | Change Statistics | | | | |
|  |  |  |  |  | R Square Change | F Change | df1 | df2 | Sig. F Change |
| 1 | .859^a^ | .739 | .714 | .23400 | .739 | 30.396 | 4 | 43 | <.001 |
| a. Predictors: (Constant), Injury level, gender, HR, Watt | | | | | | | | | |

| **Coefficients^a^** | | | | | | |
| --- | --- | --- | --- | --- | --- | --- |
| Model 4 | | Unstandardized Coefficients | | Standardized Coefficients | t | Sig. |
|  |  | B | Std. Error | Beta |  |  |
| 1 | (Constant) | .543 | .194 |  | 2.805 | .008 |
|  | Watt | .023 | .006 | .508 | 3.804 | <.001 |
|  | Heart rate | -.005 | .002 | -.255 | -2.732 | .009 |
|  | Gender | .308 | .083 | .323 | 3.712 | <.001 |
|  | Injurylevel | .247 | .074 | .409 | 3.359 | .002 |
| a. Dependent Variable: Measured VO_2peak_ (L/min) | | | | | | |

| **Correlations** | | | | |
| --- | --- | --- | --- | --- |
| Sample 4 | | | Measured VO_2peak_ (L/min) | Predicted sample 4 |
| .00 | Measured VO_2peak_ (L/min) | Pearson Correlation | 1 | .929^**^ |
|  |  | Sig. (2-tailed) |  | <.001 |
|  |  | N | 14 | 14 |
|  | Predicted sample 4 | Pearson Correlation | .929^**^ | 1 |
|  |  | Sig. (2-tailed) | <.001 |  |
|  |  | N | 14 | 14 |
| 1.00 | Measured VO_2peak_ (L/min) | Pearson Correlation | 1 | .859^**^ |
|  |  | Sig. (2-tailed) |  | <.001 |
|  |  | N | 48 | 48 |
|  | Predicted sample 4 | Pearson Correlation | .859^**^ | 1 |
|  |  | Sig. (2-tailed) | <.001 |  |
|  |  | N | 48 | 48 |
| **. Correlation is significant at the 0.01 level (2-tailed). | | | | |

**Sample 5**

| **Model 5 Summary** | | | | | | | | | |
| --- | --- | --- | --- | --- | --- | --- | --- | --- | --- |
| Model | R | R Square | Adjusted R Square | Std. Error of the Estimate | Change Statistics | | | | |
|  |  |  |  |  | R Square Change | F Change | df1 | df2 | Sig. F Change |
| 1 | .872^a^ | .761 | .740 | .22720 | .761 | 36.656 | 4 | 46 | <.001 |
| a. Predictors: (Constant), Injury level, gender, HR, Watt | | | | | | | | | |

| **Coefficients^a^** | | | | | | |
| --- | --- | --- | --- | --- | --- | --- |
| Model 5 | | Unstandardized Coefficients | | Standardized Coefficients | t | Sig. |
|  |  | B | Std. Error | Beta |  |  |
| 1 | (Constant) | .572 | .168 |  | 3.402 | .001 |
|  | Watt | .022 | .005 | .534 | 3.957 | <.001 |
|  | Heart rate | -.005 | .002 | -.300 | -3.161 | .003 |
|  | Gender | .347 | .084 | .324 | 4.129 | <.001 |
|  | Injury level | .275 | .072 | .441 | 3.793 | <.001 |
| a. Dependent Variable: Measured VO_2peak_ (L/min) | | | | | | |

| **Correlations** | | | | |
| --- | --- | --- | --- | --- |
| Sample 5 | | | Measured VO_2peak_ (L/min) | Predicted sample 5 |
| .00 | Measured VO_2peak_ (L/min) | Pearson Correlation | 1 | .946^**^ |
|  |  | Sig. (2-tailed) |  | <.001 |
|  |  | N | 11 | 11 |
|  | Predicted sample 5 | Pearson Correlation | .946^**^ | 1 |
|  |  | Sig. (2-tailed) | <.001 |  |
|  |  | N | 11 | 11 |
| 1.00 | Measured VO_2peak_ (L/min) | Pearson Correlation | 1 | .872^**^ |
|  |  | Sig. (2-tailed) |  | <.001 |
|  |  | N | 51 | 51 |
|  | Predicted sample 5 | Pearson Correlation | .872^**^ | 1 |
|  |  | Sig. (2-tailed) | <.001 |  |
|  |  | N | 51 | 51 |
| **. Correlation is significant at the 0.01 level (2-tailed). | | | | |

Scatter plots showing measured and predicted VO_2_peak for each model; full model and 5 samples.


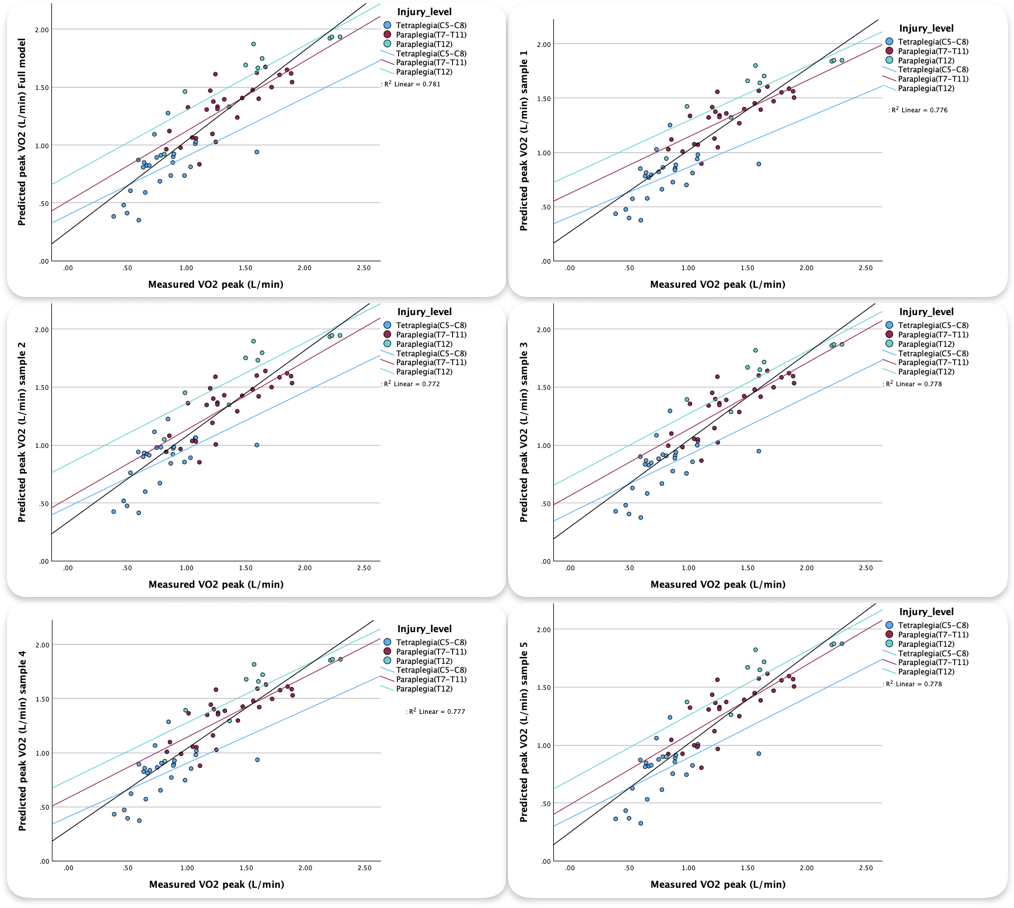

Supplement: S2 File — (DOCX) [file pone.0344188.s002.docx]
